# Liver metastatic basaloid squamous cell carcinoma with negative expression of pancytokeratin: a case report and literature review

**DOI:** 10.1186/s13000-019-0881-6

**Published:** 2019-09-06

**Authors:** Linxiu Liu, Xuemin Xue, Liyan Xue

**Affiliations:** Department of Pathology, National Cancer Center/National Clinical Research Center for Cancer/Cancer Hospital, Chinese Academy of Medical Sciences and Peking Union Medical College, Beijing, 100021 China

**Keywords:** Esophageal, Basaloid squamous cell carcinoma, Metastasis, Negative expression of pancytokeratin

## Abstract

**Background:**

Basaloid squamous cell carcinoma (BSCC) is a rare subtype of squamous cell carcinoma with a high rate of distant metastasis. BSCC occurs most commonly in the esophagus, lungs, and head and neck. However, BSCC occurring in an atypical site without a known primary tumor and/or with the presence of atypical immunohistochemical features can result in delayed diagnosis or misdiagnosis.

**Case presentation:**

Here, we report a case of a 67-year-old man with liver metastatic BSCC with negative pancytokeratin (AE1/AE3) expression. He presented with a chief complaint of epigastric discomfort. Imaging examination revealed a subcapsular mass in the right anterior lobe of the liver. Then, the patient underwent an irregular right hepatectomy. Grossly, the mass was gray, with a size of 7 × 7 × 4 cm. Microscopically, the mass comprised epithelioid tumor cells with both solid and pseudoadenoid structures, accompanied by necrosis. Immunohistochemical staining showed that the tumor cells were negative for AE1/AE3, CK18, CK7, CK19, Hepatocyte Paraffin-1, Glypican-3, Arginase-1, CD56, Chromogranin A, Synaptophysin, Vimentin, and Carcinoembryonic antigen. The Ki-67 index was 80%.The mass was diagnosed as a malignant tumor but could not be classified further. One month after surgery, the patient’s reexamination revealed esophageal tumor, and biopsy revealed BSCC. The slides of the liver tumor were reviewed, and the morphology was similar to that of the esophageal tumor. Moreover, supplementary immunohistochemical staining of liver tumor indicated p63 and p40 were strongly positive, that confirmed the liver tumor was metastatic BSCC. Previous studies have reported that 3.7% of esophageal BSCCs did not express AE1/AE3.

**Conclusion:**

When a malignant tumor comprises epithelioid cells with solid and/or pseudoadenoid structures, but not adenocarcinoma or neuroendocrine carcinoma, even if the tumor cells are negative or weakly positive for AE1/AE3, we should consider BSCC. For a definite diagnosis, immunohistochemical staining for squamous cell carcinoma markers, including p63 and p40, and examination of common primary sites of BSCC should be performed.

## Background

Basaloid squamous cell carcinoma (BSCC) is a rare and special subtype of squamous cell carcinoma. BSCC usually occurs in older men and most commonly in the esophagus, lungs, and head and neck. The diagnosis of BSCC mainly depends on its histological morphological features [1–3]. It has been never reported there is primary BSCC in the liver. However, there are certain cases in which BSCC of the liver is found first, and then the primary tumor is found or arduous to discover [4]. BSCC could present a pseudoadenoid pattern, and the immunohistochemical markers of squamous cell carcinoma, which include p63, p40 and CK5/6, could serve to confirm the diagnosis. BSCC has shown high positivity for pancytokeratin (AE1/AE3), but in rare cases, this expression is absent [5, 6]. When epithelioid malignancy in the liver or other sites presents a pseudoadenoid structure, especially with AE1/AE3 negative expression, the possibility of metastatic BSCC may be rarely considered without a history of primary cancer, which results in delayed diagnosis or misdiagnosis. Here, we report a case of liver metastatic BSCC with negative expression of AE1/AE3 and reviewed the relevant literature.

## Case presentation

A 67-year-old man presented to our hospital with the chief complaint of epigastric discomfort for a few months. There was no history of hepatitis or tumor. Magnetic resonance imaging (MRI) of the liver showed solitary hepatic space-occupying lesions with a size of approximately 5.2 × 4.2 cm, mainly located under the capsule of the right liver V and VI segments. Abdominal ultrasonography revealed a heterogeneous echogenic mass in the right lobe of the liver, which was 7.5 × 9.3 cm in size and located under the liver capsule. There was no imaging examination other than the normal frontal and lateral chest examinations. Then, the patient underwent irregular resection of the right liver, and a hilar lymph node dissection was performed. During the operation, there were no ascites, and the liver was dark red without obvious cirrhosis.

**Fig. 1:**
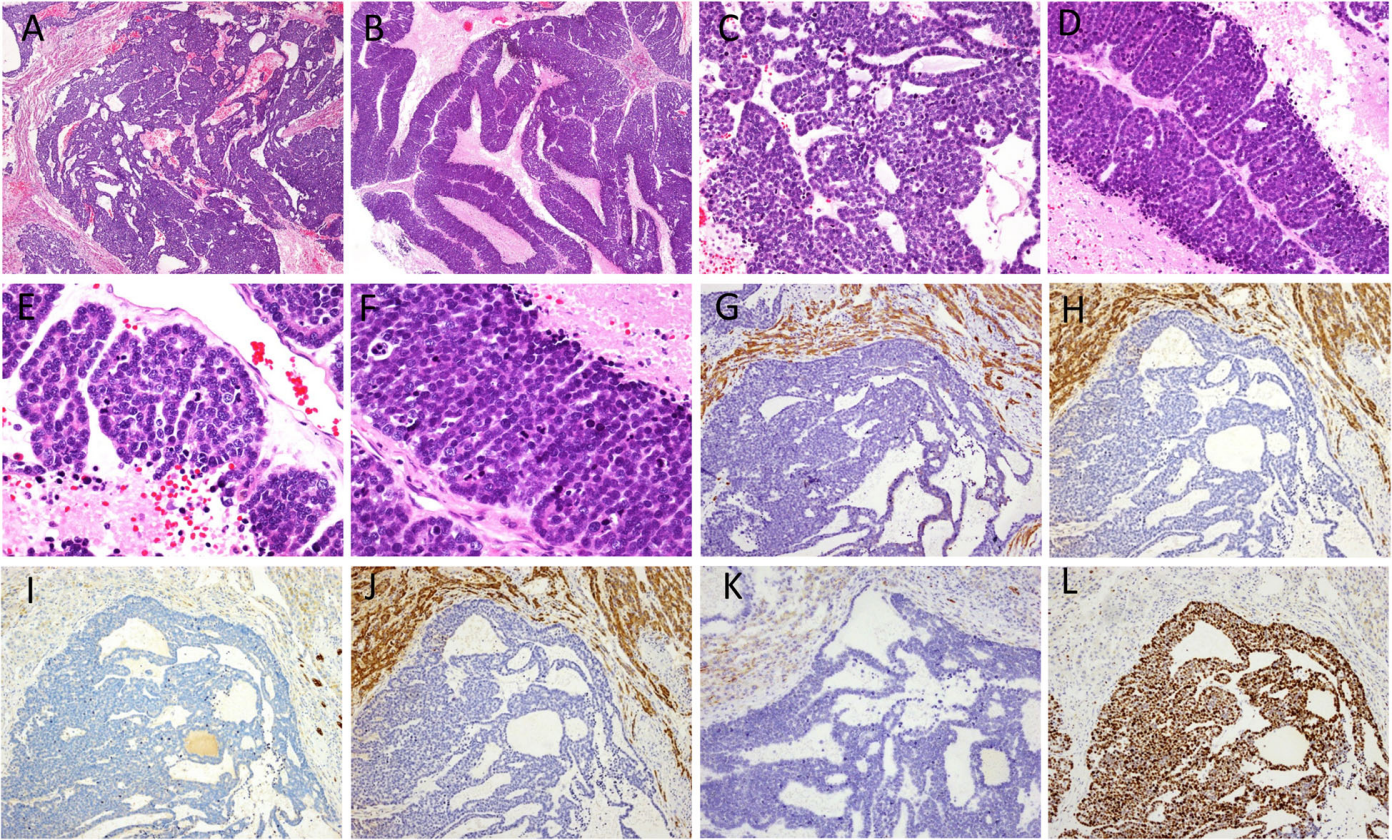
Pathological findings of the liver tumor. **a** The hepatic tumor was composed of monotonous epithelioid cells (hematoxylin and eosin [H&E], 40×). **b** (H&E, 40×) and **c** (H&E, 200×) The tumor cells were closely arranged with solid and pseudoadenoid structures. **d** Tumor necrosis was obvious (H&E, 200×). **e** and **f** The tumor cells were round or ovoid, with hyperchromatic nuclei, scant basophilic cytoplasm, and increased mitotic activity (H&E, 400×). Immunohistochemical staining showed that the tumor cells were negative for AE1/AE3 (**g**), CK18 (**h**), CK7 (**i**), Hep Par-1 (**j**), and CD56 (**k**). The Ki-67 index was 80% (**l**) (40×)

Grossly, the mass was 7 × 7 × 4 cm in size. The mass was fragile and gray. Histopathological examination revealed that the tumor was composed of monotonous epithelioid cells (Fig. 1a), which were closely arranged with solid and pseudoadenoid structures (Fig. 1b and c). Tumorous necrosis was obvious (Fig. 1d). The tumor cells were round or ovoid, with hyperchromatic nuclei, scant basophilic cytoplasm, and increased mitotic activity (Fig. 1e and Fig. 1f). Immunohistochemical staining showed that the tumor cells were negative for AE1/AE3 (Fig. 1g), CK18 (Fig. 1h), CK7 (Fig. 1i), Hepatocyte Paraffin-1(Hep Par-1) (Fig. 1j), Glypican-3 (GPC-3), Arginase-1 (ARG-1), CD56 (Fig. 1k), Chromogranin A (CgA), Synaptophysin (Syn), Vimentin, and Carcinoembryonic antigen (CEA). The Ki-67 index was 80% (Fig. 1l). Due to atypical histological morphology and immunohistochemical characteristics, the mass was diagnosed as a malignant tumor but could not be classified further.

**Fig. 2:**
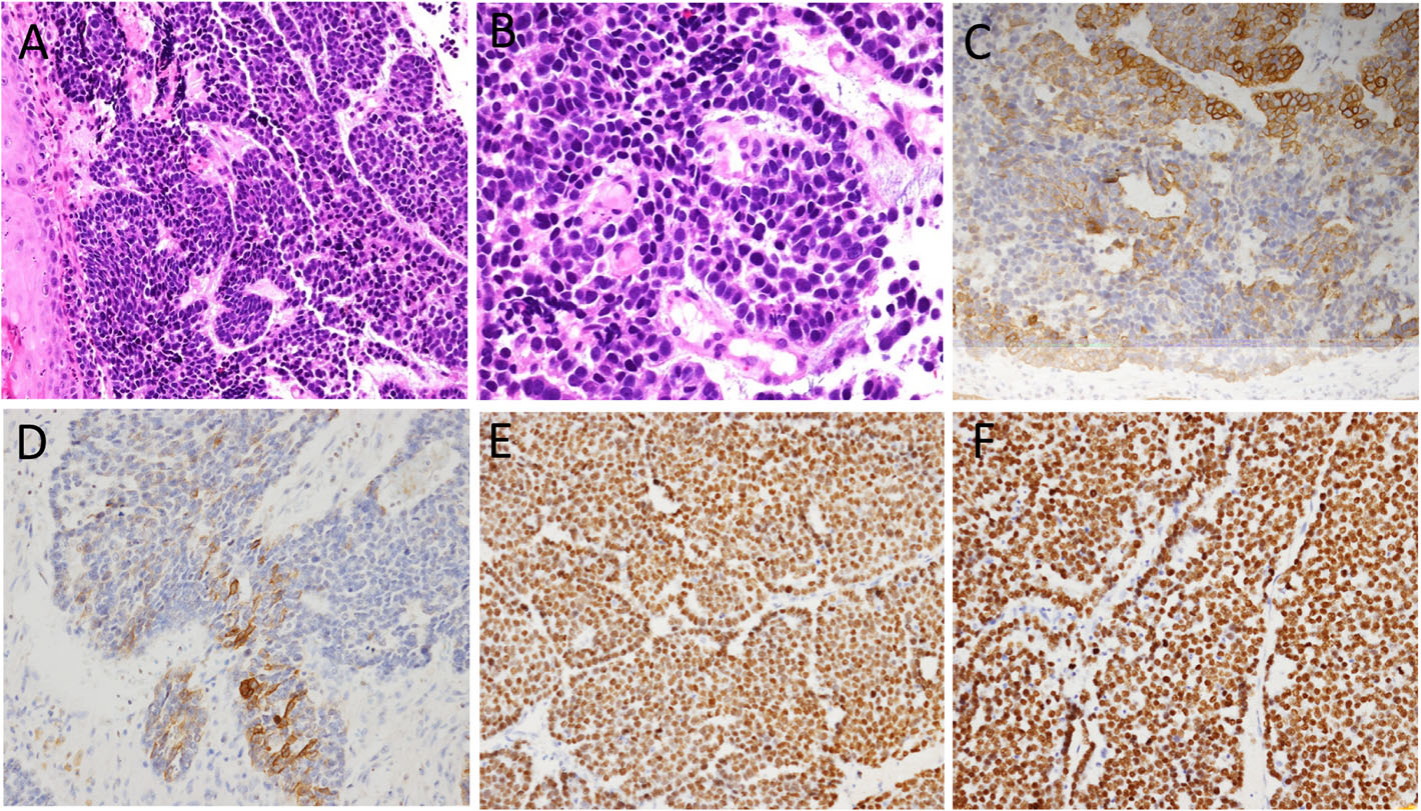
Pathological findings of the esophageal tumor and additional immunohistochemical staining of the liver tumor. **a** (H&E, 200×) and **b** (H&E, 400×). Histological morphology of esophageal tumor. **c** Esophageal tumor was weakly reactive to AE1/AE3 (200×). The liver tumor cells were weakly reactive to CK5/6 (**d**) but diffusely positive for p40 (**e**) and p63 (**f**) (200×)

One month after surgery, the patient underwent computed tomography (CT) scan, which revealed that the middle thoracic esophageal wall was thickening. Endoscopic examination showed an esophageal tumor, and biopsy revealed BSCC (Fig. 2a and b). The esophageal tumor cells were weakly reactive to AE1/AE3 (Fig. 2c) and strongly reactive to CK5/6, p40 and p63. Then, slides of the liver tumor were reviewed, and the morphology was similar to that of an esophageal tumor. Moreover, supplementary immunohistochemistry staining of the liver tumor, including CK5/6, p40 and p63, was performed. The liver tumor cells were weakly reactive to CK5/6 (Fig. 2d) and diffusely positive for p40 (Fig. 2e) and p63 (Fig. 2f), which proved that the liver tumor was metastatic BSCC.

The patient died in 3 months after conservative treatment without further surgical treatment, radiotherapy or chemotherapy because he was in poor physical condition.

## Discussion

BSCC is prone to distant metastasis. The common organs of distant metastasis include the lungs, liver and bone [1, 6]. The histological morphology mainly included (1) multiple invasive growth modes, such as solid, lobular, cribriform, pseudoadenoid, trabecular, etc., accompanied by central comedonecrosis; (2) tumor cells with hyperchromatic nuclei, scant basophilic cytoplasm, and increased mitotic activity; (3) palisade tumor cells surrounding the nests; (4) basement membrane-like substances between the cells; and (5) with or without common squamous cell carcinoma components. BSCC is usually positive for immunohistochemical staining of epithelial markers, including AE1/AE3, CK5/6, CK14, CK34βE12, p63 and p40. A few cases showed the weak expression of neuroendocrine markers. A portion of the BSCC of the head and neck and BSCC of the anus are associated with human papillomavirus (HPV) infection, and p16 is positive [2, 5, 7–10]. However, BSCCs of esophagus have shown zero positivity rates for HPV DNA, as assessed in situ hybridization (ISH) [11].

The diagnosis of BSCC is mainly based on its histological morphology, so we should be familiar with its morphology features. However, it is difficult to consider the diagnosis of metastatic tumors, if without known primary BSCC history. The primary tumor may be occult. Bastiaan DBW et al. [4] reported a case of liver BSCC with characteristic histological morphology. The patient died of extensive abdominal and pelvic metastasis without a primary lesion found in 2 years, and no autopsy was performed after death. The authors suggested that although no other primary tumor has been diagnosed, it is not possible to prove that this is a primary liver tumor without autopsy to rule out occult malignancies elsewhere. The case we reported herein had no known primary tumor before surgery. Unfortunately, we did not diagnosed as metastatic BSCC until the primary esophageal tumor was found by chance. In fact, the morphology of the liver tumor was typical BSCC. Thus, clinical physical examination, imaging examination and endoscopic examination are necessary to rule out metastatic tumors from other sites.

The confusion in this case resulted from the fact that the expression of cytokeratin, including AE1/AE3, CK18 and CK7, was negative in the metastatic site. This finding led us to diagnose this case as a malignant tumor, but not a carcinoma. Thus, the pertinent literature was reviewed. No studies on the immunohistochemical staining of AE1/AE3 in metastatic BSCC and no negative results were reported. However, in the study of primary BSCCs, 108 esophageal BSCCs with AE1/AE3 immunohistochemical staining were reviewed. Four were negative, with a negative rate of 3.7% [6, 12–17] (Table 1). And negative AE1/AE3 expression was also found in BSCC of head and neck [18]. In addition to the negative expression of AE1/AE3, there are also cases in which CK5/6 is not expressed in BSCC. Patil DT et al. [19] reported that 3 of 15 cases were negative for CK5/6 in BSCC of the anal canal. Cho k et al. [20] reported that 9 out of 26 patients with BSCC of the head and neck did not express CK5/6. However, there is no literature reporting that BSCC lacks the expression of p40 and p63, which may be more effective markers for BSCC.

**Table 1:** Immunohistochemical staining results for AE1/AE3 in primary esophageal BSCC

References	Publication year	Cases of BSCC	Cases negative for AE1/AE3
[6]	2013	59	2
[12]	2000	10	0
[13]	2001	1	0
[14]	2001	1	0
[15]	2003	5	0
[16]	2006	15	0
[17]	1998	16	2
This case^a^		1	0^a^
Total		108	4

^a^In this case, the primary lesion was weakly positive for AE1/AE3, while the metastatic lesion was negative

## Conclusion

Pathologists should be well aware of the histological and morphological features of BSCC. In the uncommon locations of BSCC, where the malignant tumor is composed of epithelioid cells with solid and/or pseudoadenoid structures, but not adenocarcinoma or neuroendocrine carcinoma, even if the tumor cells are negative or weakly positive for pancytokeratin, we should consider BSCC. For a definite diagnosis, immunohistochemical staining for squamous cell carcinoma markers and the examination of common primary sites of BSCC should be performed.

## Data Availability

As a case report, all data generated or analyzed are included in this article.

## Abbreviations

AE1/AE3: pancytokeratin
AFP: Alpha-fetoprotein
ARG-1: Arginase-1
BSCC: Basaloid squamous cell carcinoma
CEA: Carcinoembryonic antigen
CgA: Chromogranin A
CT: Computed tomography
GPC-3: Glypican-3
H&E: Hematoxylin and eosin
Hep Par-1: Hepatocyte Paraffin-1
HPV: Human papillomavirus
syn: synaptophysin

## References

[1] Wain SL, Kier R, Vollmer RT, Bossen EH (1986). Basaloid-squamous carcinoma of the tongue, hypopharynx, and larynx: report of 10 cases. Hum Pathol.

[2] Cho K (2010). Basaloid squamous cell carcinoma of the upper Aerodigestive tract. Korean J Pathol.

[3] Zhou L (2015). Pathological analysis of basaloid squamous cell carcinoma of the esophagus. China J Clin Ration Drug Use.

[4] Bastiaan DBW (2000). Basaloid squamous carcinoma in the liver. Pathology.

[5] Wenig BM (2017). Squamous cell carcinoma of the upper aerodigestive tract: dysplasia and select variants. Mod Pathol.

[6] Zhang BH, Cheng GY, Xue Q, Gao SG, Sun KL, Wang YG, Mu JW, He J (2013). Clinical outcomes of basaloid squamous cell carcinoma of the esophagus: a retrospective analysis of 142 cases. Asian Pac J Cancer Prev.

[7] Imamhasan A, Mitomi H, Saito T, Hayashi T, Takahashi M, Kajiyama Y, Yao T (2012). Immunohistochemical and oncogenetic analyses of the esophageal basaloid squamous cell carcinoma in comparison with conventional squamous cell carcinomas. Hum Pathol.

[8] Villada G, Kryvenko ON, Campuzano-Zuluaga G, Kovacs C, Chapman J, Gomez-Fernandez C (2016). A limited Immunohistochemical panel to distinguish basal cell carcinoma of cutaneous origin from basaloid squamous cell carcinoma of the head and neck. Appl Immunohistochem Mol Morphol.

[9] Shroyer KR, Brookes CG, Markham NE, Shroyer AL (1995). Detection of human papillomavirus in anorectal squamous cell carcinoma. Correlation with basaloid pattern of differentiation. Am J Clin Pathol.

[10] Graham RP, Arnold CA, Naini BV, Lam-Himlin DM (2016). Basaloid squamous cell carcinoma of the anus revisited. Am J Surg Pathol.

[11] Bellizzi AM, Woodford RL (2009). Christopher. Basaloid squamous cell carcinoma of the esophagus: assessment for high-risk human papillomavirus and related molecular markers. Am J Surg Pathol.

[12] Ni XH, Yu GH, Zhang G, Wu MJ, Shao LH (2000). Esophageal basaloid squamous carcinoma: a clinicopathologic and immunohistochemical study of 10 cases. Pract J Cancer.

[13] Nishimura W, Naomoto Y, Hamaya K, Toda S, Miyagi K, Tanaka N (2001). Basaloid-squamous cell carcinoma of the esophagus: diagnosis based on immunohistochemical analysis. J Gastroenterol Hepatol.

[14] Kawahara K, Makimoto K, Maekawa T, Yamamoto S, Shiraishi T, Takahashi S, Shirakusa T, Nakayama Y, Kikuchi M (2001). An immunohistochemical examination of basaloid squamous cell carcinoma of the esophagus: report of a case. Surg Today.

[15] Cao LY, Sun XH (2003). A histochemical and immunohistochemical observation of basaloid squamous cell carcinoma in the esophagus. Chin J Clin Oncol.

[16] Li WY, NIU FX, Li QY. Basaloid squamous carcinoma of the esophagus immunohistochemical, light and electron microscopic. Cancer Res Prev Treat. 2006;33(07):521–23.

[17] Zhang XH, Sun GQ, Zhou XJ, Guo HF, Zhang TH (1998). Basaloid squamous carcinoma of esophagus: a clinicopathological, immunohistochemical and electron microscopic study of sixteen cases. World J Gastroenterol.

[18] Banks Evelyn R., Frierson Henry F., Mills Stacey E., George Evan, Zarbo Richard J., Swanson Paul E. (1992). Basaloid Squamous Cell Carcinoma of the Head and Neck. The American Journal of Surgical Pathology.

[19] Patil DT, Goldblum JR, Billings SD (2013). Clinicopathological analysis of basal cell carcinoma of the anal region and its distinction from basaloid squamous cell carcinoma. Mod Pathol.

[20] Cho K, Jeong SU, Kim SB, Lee S, Choi S, Nam SY, Kim SY (2017). Basaloid squamous cell carcinoma of the head and neck: subclassification into basal, ductal, and mixed subtypes based on comparison of Clinico-pathologic features and expression of p53, cyclin D1, epidermal growth factor receptor, p16, and human papillomavirus. J Pathol Transl Med.

